# Population mixing mediates the intestinal flora composition and facilitates invasiveness in a globally invasive fruit fly

**DOI:** 10.1186/s40168-023-01664-1

**Published:** 2023-09-28

**Authors:** Yidan Wang, Zhihong Li, Zihua Zhao

**Affiliations:** Department of Plant Biosecurity & MARA Key Laboratory of Surveillance and Management for Plant Quarantine Pests, College of Plant Protection, China Agricultural University, Beijing, 100193 China

**Keywords:** *Bactrocera dorsalis*, Inbred, Outbred, Phenotype, Microbiome, Transcriptome

## Abstract

**Background:**

Changes in population heterozygosity and genetic diversity play important roles in mediating life history traits of organisms; these changes often lead to phenotypic evolution in offspring, which become superior to their parents. In the present study, we examined phenotypic differentiation, the intestinal microbiome composition, and metabolism shift in the oriental fruit fly (*Bactrocera dorsalis*) by comparing an inbred (monophyletic) original population and an outbred (mixed) invasive population.

**Results:**

The results showed that the outbred population of *B. dorsalis* had significantly higher biomass, adult longevity, and fecundity than the inbred population. Additionally, intestinal microflora analysis revealed that both *Diutina rugosa* and *Komagataeibacter saccharivorans* were significantly enriched in the outbred population with higher genetic heterozygosity. *D. rugosa* enrichment altered amino acid metabolism in the intestinal tract, and supplementing essential amino acids (e.g. histidine and glutamine) in the diet led to an increase in pupal weight of the outbred population. Additionally, transcriptome analysis revealed that the HSPA1S gene was significantly downregulated in the outbred population. HSPA1S was involved in activation of the JNK-MAPK pathway through negative regulation, caused the upregulation of juvenile hormone (JH), and led to an increase in biomass in the outbred flies.

**Conclusion:**

In conclusion, the outbred population had an altered intestinal microbe composition, mediating metabolism and transcriptional regulation, leading to phenotypic differentiation; this may be a potential mechanism driving the global invasion of *B. dorsalis*. Thus, multiple introductions could lead to invasiveness enhancement in *B. dorsalis* through population mixing, providing preliminary evidence that changes in the intestinal microbiome can promote biological invasion.

Video Abstract

**Supplementary Information:**

The online version contains supplementary material available at 10.1186/s40168-023-01664-1.

## Background

The successful invasion of an alien organism often leads to adaptive colonization, continuous reproduction, and the expansion of invasive populations, which seriously threaten agriculture, forestry, and animal husbandry worldwide and are extremely harmful to economic development [1, 2]. The invasive alien species (IAS) could compete with local species or protected species for ecological niches and, in serious cases, lead directly or indirectly to a reduction in/loss of native biodiversity in the invaded area, adversely deteriorating ecosystem functions [3–5].

It is generally believed that high levels of genetic diversity facilitate the establishment and persistence of IAS in new habitats [6]. For example, high genetic diversity in *Arabidopsis thaliana* can speed up the initial emergence and flowering time of seedlings and increase biomass and reproductive capacity [7, 8]. Furthermore, hybrid progeny may enhance their phenotype and invasiveness due to the high genetic diversity caused by increasing heterozygosity [9]. For example, locally grown *Spartina maritima* in the UK was crossed with introduced *Spartina alterniflora* to form allotetraploid *Spartina anglica*, which had greatly enhanced invasiveness, resulting in displacement of the parent strains in coastal areas and rapid colonization and spread to many new areas [10, 11].

Given the strong link between species introductions and global trade and transport, it is not surprising that many IAS are introduced from several sources. The propagule pressure of IAS has been well explored and is related to the bottleneck effect, Allee effects, and founder effect. Multiple introductions (e.g. of the oriental fruit fly) from different geographical populations could provide opportunities for intraspecific hybridization and the evolution of new genotypes [6, 12, 13]. The genetic admixture of different geographical populations of IAS could directly affect the fitness of colonies through heterosis after intraspecific hybridization, which is an unexplored topic in invasion ecology [14–16]. It can also greatly enhance the adaptive potential of colonizing populations by subjecting new allelic combinations to natural selection [6, 16–20]. For example, the invasive population of the pine wood nematode, *Bursaphelenchus xylophilus*, in China originated from many different countries [21]. Maintaining high genetic diversity during the invasion process is one of the invasion mechanisms of successful IAS [21, 22].

Some mechanisms, such as gene recombination and invasion evolution acting on individual traits and trait combinations, have been shown to drive the production of key genetic characteristics after interspecific hybridization [18, 23]. In the context of intraspecific hybridization, genetic admixture has been shown to greatly increase genetic phenotypic variation in invasive populations of plants [24]. For example, the grain weight per panicle and grain yield in a millet hybrid population were higher than those in their parents [25]. Heterosis in fecundity, growth traits, and genetic diversity of *Macrobrachium rosenbergii* has been observed [26]. In recent years, gut microbes and their symbiotic functional genomes have been important mechanisms of enhanced invasiveness in hybrid offspring [27, 28]. For example, all hybrid offspring of two carp species widely distributed in the USA, bighead carp, *Hypophthalmichthys nobilis*, and silver carp, *Hypophthalmichthys molitrix*, have high fertilization rates and high embryo viability [27].

The oriental fruit fly (OFF, *Bactrocera dorsalis*) is one of the most important and notorious pests worldwide, with the widest host range; it causes major losses in the global fruit and vegetable industries. Previous research revealed that the invasive population of *B. dorsalis* originated from several geographical populations (e.g. North China) [29, 30]. For example, the molecular marker mitochondrial CO1 clearly showed that the invasive population in North China was a mixed population originating from tropical (Southeast Asia) and temperate regions (South China) [31, 32]. The invasions of *B. dorsalis* in California, USA, were also due to multiple, independent introductions with a wide variety of origins (e.g. from areas in Africa, Southeast Asia, and China), in addition to the previously known source in Hawaii [33, 34]; these introductions led to an invasive mixed population resulting from several geographical populations. In our previous research, we found that the δ^2^H stable isotope values from *B. dorsalis* from Beijing were not consistent with those in Beijing rainwater but rather were consistent with the data from Fuzhou in southeast China [35]. Furthermore, the evidence of both stable isotope and molecular markers indicated that the invasive population in North China was an outbred population resulting from the mixture of several geographical populations in South China [35]. Thus, this mixed outbred population represents the invasive population of *B. dorsalis*, which is an unexplored field in biological invasion [30, 36]. Therefore, the invasive population of *B. dorsalis* comprises hybrid offspring of individuals from several geographical populations; this could increase the invasiveness of *B. dorsalis* and cause a major threat to agriculture [37, 38]. We addressed two research topics in the present research: (i) phenotypic differences between original (inbred) and invasive (outbred) populations of *B. dorsalis* were determined to reveal invasiveness traits by using phenotypic omics analyses, and (ii) multiomics (phenomics, microbiomics, amino acid mics, transcriptomics) technology was used to examine holobiont and microbiome interactions and explore the potential invasion mechanism of the global invasive *B. dorsalis*. We hope that these findings will be helpful in guiding prevention and control of *B. dorsalis* worldwide.

## Methods

### Inbred and outbred populations

**Fig. 1:**
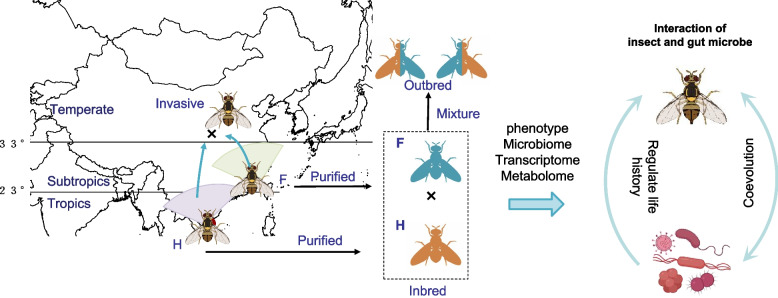
Diagram of experimental design for the inbred and outbred populations of the oriental fruit fly (*Bactrocera dorsalis*)

South Asia and Southeast Asia are the major distribution centres of the oriental fruit fly, *Bactrocera dorsalis* [13, 39]. In China, *B. dorsalis* is mainly distributed in tropical and subtropical regions of the south part [4, 40]. To obtain the inbred population, we separately collected three wild-caught populations from two geographical locations (three replicates from Fujian (F) province and three replicates from Hainan (H) province) in citrus orchards in 2018 (Fig. 1). Then, all wild-caught populations of flies were reared on artificial diets [4, 41]. The F1 flies of all six wild-caught populations (three from F and three from H) were reared under the same conditions (temperature: 28 ± 1 °C, light:dark photoperiod: 14 h:10 h, and humidity: 65 ± 5%). For each wild-caught population, 10 pairs of F1 adult flies were randomly selected to conduct purification to obtain the inbred population. We used the same procedure to purify the inbred lines for 3 generations. Then, we obtained six independently established lines from the two inbred populations (each with three independent replicates). Following the same experimental format as the “inbred” populations, we combined 100 males/females of the inbred F population with 100 females/males of the inbred H population and allowed them to hybridize, forming mixed (outbred) populations (F♀ × H♂ and H♀ × F♂: Fig. 1). Then, we also obtained six outbred populations, three from female parents of the inbred F population and three from female parents of the inbred H population (Fig. 1). After that, we compared the phenotypic differences between the inbred and outbred populations.

Both the inbred and outbred populations of *B. dorsalis* were reared in an intelligent climate chamber with a 14 h:10 h light:dark photoperiod, a temperature of 28 ± 1 °C, and humidity at 65 ± 5%. After eclosion, the adult flies of each population were placed in a mesh cage and fed an artificial diet (sucrose:soy peptone = 3:1) (Supplementary Information Table 1). Water was continuously provided via a wetted cotton ball. Larvae that hatched from the collected eggs were fed an artificial diet (Supplementary Information Table 1) [41]. When the larvae reached the final larval stage, they were removed with forceps and placed in sterile sand with 60~70% water content. After pupation, the larvae were placed into a cage for subsequent feeding.

We also conducted fruit infestation experiments to examine the fecundity differences between the original and mixed (invasive) outbred populations before the experiments. Mangos were used to evaluate the infestation performance of original (F and H) and mixed outbred populations in the laboratory. We placed mangos in a fly cage containing 10 pairs of wild-caught flies to test the fecundity differences. We also determined differences in pupal weight, ovary size, and hatching rate between the original and mixed outbred populations.

### Life-history traits

#### Eggs, larvae, and pupae

One-hundred eggs were incubated on wet filter paper, and the hatch rates of eggs at 24 h, 36 h, 48 h, and 72 h were observed, respectively. Then, hatched 1st instar larvae were individually fed in a 3.5-mm petri dish filled with feed. The developmental stage of each larva was observed every 24 h, and all larval deaths were recorded. The cephalopharyngeal skeleton of 3rd instar larvae was dissected to examine differences between the inbred and outbred groups. When the larvae were reached the final larval stage, 300 individuals were selected and allowed to pupate, and pupal weight and pupation and eclosion rates were measured on the 6th day.

#### Adult survival and reproduction

The cohort of fly populations (300 newly emerged adults) were maintained to record the number of surviving individuals every day for 60 days; these data were used to calculate the survival rate, sex ratio, and pupal weight. Furthermore, 10 males/females were selected to examine their fecundity capacity. Eggs were collected from mango slices every 24 h for 20 days, and the cumulative number of eggs laid per female was counted. Additionally, the ovaries of female adults were dissected to determine their size under the microscope on days 5 and 15. Survival and reproduction data were used to calculate the parameters of the life table [30, 42, 43].

### Omics analyses

#### Intestinal dissection and microbiome composition

Final-stage larvae (30 individuals) of the inbred (H and F) and outbred (F♀ × H♂ and H♀ × F♂) populations were dissected to analyse composition of the intestinal microbiome. All samples were stored on dry ice and sent to Shanghai Applied Protein Technology Co., Ltd. for metabolic analysis of amino acids and derivatives and to Shanghai Majorbio Biopharm Technology Co., Ltd. for microbial diversity sequencing analysis and transcriptome sequencing (Supplementary Information for Methods).

Bacterial amplification was performed in intestinal samples from the inbred and outbred populations on the Illumina HiSeq platform using the 16S rDNA ITS1 variable region and 16S rDNA V3–V4 variable region, respectively. First, data quality was controlled and screened. Next, the diversity index was analysed according to operational taxonomic unit (OTU) cluster analysis and species taxonomic analysis. Biostatistical analysis was performed to understand the relative species abundance of bacteria and fungi in the samples, and linear discriminant analysis effect size (LEfSe) was used to evaluate the significance of species richness differences. Finally, the intestinal microbes with significant differences between the inbred and inbred groups were identified.

The effects of different bacterial groups on pupal weight were verified by exchanging the diet provided to the inbred and outbred populations. We fed larvae of outbred populations a spent diet (i.e. food that had already been eaten for 3 days by larvae of inbred populations). Similarly, the larvae of inbred populations were fed a spent diet that had been eaten for 3 days by larvae of outbred populations. We exchanged the spent diets of the outbred populations and inbred population to examine the influence of gut microbiome exchange on the life history of flies. Also, the effects of differential amino acids on pupal weight were verified by adding histidine, arginine, glutamine, glutamate, isoleucine, and valine to the artificial diet of an inbred population (see Supplementary Information for Methods). We also compared the phenotypic differences and intestinal microflora between inbred and outbred populations from the same female source, which could examine maternal effects of inbred population on the intestinal microflora of the hybridized flies of outbred population.

### Transcriptome analysis

Heterozygosity between the inbred and outbred populations was represented by the number of single-nucleotide polymorphisms (SNPs) measured by transcriptomic analysis. Raw data were qualitatively controlled, optimized, and evaluated using SeqPrep, and gene/transcript expression levels were quantitatively analysed using RSEM. Then, DESeq2 was used to analyse the different expression levels of genes between samples to identify the differentially expressed genes to study the function of differentially expressed genes. The differentially expressed genes were divided into different gene sets for analysis according to the up- and downregulation of genes, and the obtained gene sets were subjected to functional enrichment analysis to obtain the main metabolic pathways.

### Statistical analysis

SPSS 25.0 was used for data analysis. The Z-score extreme value standardization method was used to process the original data, and extreme values with an absolute Z score higher than 2 were excluded. Then, the data were tested for homogeneity of variance and a normal distribution. If the *P*-value of the test for homogeneity of variance was greater than or equal to 0.05 and the data were normally distributed, one-way ANOVA or the *T*-test was used for biostatistical analysis of the data. If one of these criteria was not met, then nonparametric tests were used.

Origin 2021 was used to perform nonlinear fitting for the mortality rate and accumulated offspring quantity of *B. dorsalis* in the different populations. The fitting result had a high fitting degree with the logistic curve, so the logistic curve was used to fit the mortality rate and accumulated offspring quantity in *B. dorsalis* to determine whether outbreeding affected survival and reproduction. Then, survival parameters, such as the death distribution and death expectation, and reproductive parameters, such as oviposition distribution and oviposition expectation, were calculated using the fitted data. Origin 2021 was used to draw all the resulting graphs.

## Results

### The invasiveness of mixed population

We found that the mixed outbred population had significantly higher pupal weight (*P* < 0.001) and ovary size (*P* < 0.001) than the original population in the mangos (Supplemental Information Fig. 1 A and B). Additionally, the number of eggs laid per female per day (*P* < 0.001) and hatching rates (*P* < 0.001) were higher in the mixed outbred population than in the original F and H population (Supplementary Information Fig. 1C and D).

### Phenotypic differences between inbred and outbred OFFs

**Fig. 2:**
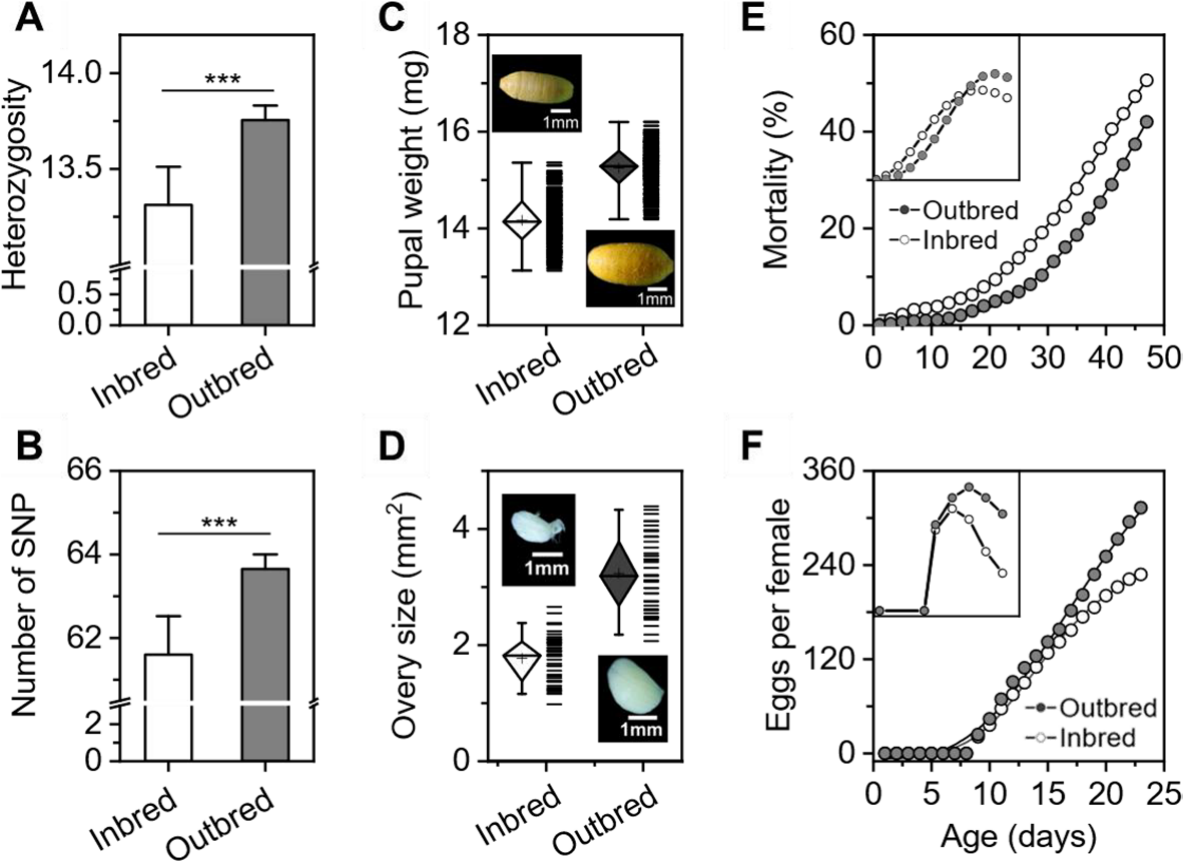
Phenotypic differences between the inbred and outbred populations. **A** Heterozygosity. **B** Number of SNPs. **C** Pupal weights; the pictures of pupae were taken under a microscope. **D** Ovary size, calculated by multiplying the length of the ovary by the width; the pictures of ovaries were also taken under a microscope. **E** Adult mortality rate between the inbred and outbred population. The small figure shows the death distribution of *B. dorsalis*. **F** Cumulative number of eggs laid by a single female. The small area shows the reproduction distribution between the inbred and outbred population

The outbred population had higher genetic heterozygosity (*F*_1.7_ = 4.324, *P* =0.33) and a larger number of SNPs (*F*_1.7_ = 4.323, *P* = 0.33) than the inbred population (Fig. 2A and B). Additionally, the outbred population had a higher pupal weight (*F*_1.156_ = 591.78, *P* < 0.001) than the inbred population, with an increase of 7.32% (Fig. 2C). However, diet intake volume and the development rate did not differ between the two populations (*F*_1.156_ = 591.78, *P* < 0.001) (Supplementary Information Fig. 2). Additionally, adult female ovaries in the outbred population were 34.47% larger than those of the inbred population at 15 days post-emergence (*F*_1.87_ = 99.45, *P* < 0.001) (Fig. 2D). The ovary length and width in the outbred population were 23.15% (*F*_1.87_ = 52.14, *P* < 0.001) and 55.02% (*F*_1.87_ = 117.74, *P* < 0.001) longer and wider than those in the inbred population, respectively.

The mortality curve of the outbred population was significantly lower than that of the inbred population (*F*_1.7_ = 64.73, *P* < 0.05) (Fig. 2E). Regarding cohort fecundity, the outbred population laid a significantly larger number of eggs per female than the inbred population (*F*_1.7_ = 12.52, *P* < 0.01), indicating that the reproductive potential was enhanced after population mixing (Fig. 2F). In total, the accumulated egg number in the outbred population was 26.12% higher than that in the inbred population (*P* < 0.01). The innate rate decreased from 0.33 ± 0.00 in the inbred population to 0.32 ± 0.01 in the outbred population, while the doubling time in the outbred population was 2.82% lower than that in the inbred population (Supplementary Information Table 2). Pharyngeal bone length (*P* = 0.174) and food intake (*P* = 0.275) showed no significant differences between the inbred population and outbred population (Supplementary Information Fig. 2A and B).

### Microbiome differences between inbred and outbred OFFs

**Fig. 3:**
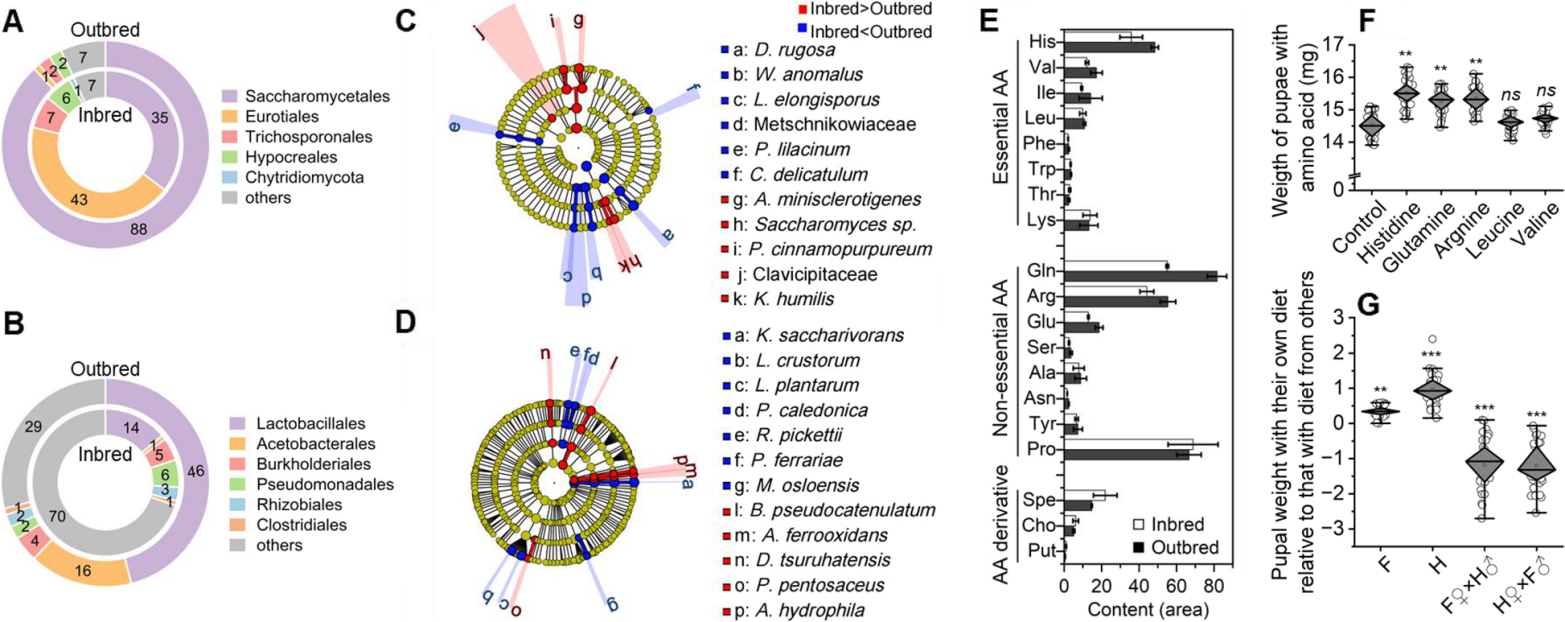
Species compositions of the microbiomes in the gut of the inbred and outbred populations. The diagram of intestinal fungal (**A**) and bacterial composition (**B**) of the intestinal flora between the inbred and outbred of *B. dorsalis*. The inner circle is the inbred population, and the outer circle is the outbred population. The LEfSe diagram of intestinal fungi (**C**) and bacteria (**D**) in *B. dorsalis*. Red indicates that the abundance of the microbe group in the inbred population was significantly higher than that in the outbred population, and the opposite is shown in blue. **E** Concentrations of amino acids and their derivatives in the intestinal tract of *B. dorsalis*. AA stands for amino acids. **F** The effects of amino acids on weight of pupae of *B. dorsalis*. **G** Pupal weight with their own diet relative to that with diet from others during the diet exchanging experiment

In total, 367,190 and 367,190 high-quality fungal and bacterial sequences were obtained, respectively. The mean base pair lengths of each sequence were 227 bp and 417 bp, respectively. The microbiome OTU analysis revealed 113 species, 67 families, and 8 phyla (Fig. 3A and B). The intestinal fungi were mainly distributed in four orders including Eurotiales (43.03%), Saccharomycetales (35.35%), Trichosporonales (7.43%), and Hypocreales (5.63%) for the inbred population. However, the intestinal fungi of the outbred population were mainly distributed in Saccharomycetales (87.95%), Trichosporonales (2.27%), Hypocreales (2.20%), and Eurotiales (1.04%). The abundance of the order Saccharomycetales in the outbred population was 148.80% higher than that in the inbred population (*P* = 0.046) (Fig. 3A). Regarding intestinal fungal LEfSe, *Diutina rugosa* (*P* = 0.009) showed the most significant difference between the outbred and inbred groups (Fig. 3C).

The number of intestinal bacteria OTUs was 2571, with 1651 species, 516 families, and 47 phyla. The intestinal bacteria in the inbred population mainly belonged to Lactobacillales (14.32%), Pseudomonadales (5.89%), Burkholderiales (4.62%), Acetobacterales (0.68%), and others (74.48%). The intestinal bacteria in the outbred population mainly belonged to Lactobacillales (45.78%), Acetobacterales (16.44%), Burkholderiales (3.64%), Pseudomonadales (2.17%), and others (31.96%). The abundance of Lactobacillales in the outbred population was 219.70% higher than that in the inbred population (*P* = 0.023). (Fig. 3B). Regarding intestinal bacteria LEfSe between the outbred and inbred groups, *Komagataeibacter saccharivorans* (*P* = 0.017) was the most significantly different between the two populations (Fig. 3D).

The concentrations of six amino acid including histidine, arginine, glutamine, glutamate, isoleucine, and valine in the intestine in the outbred population were significantly higher than those in the intestine in the inbred population (Fig. 3E). The pupal weight in the inbred population was significantly increased after adding glutamic acid and histidine to artificial diets. In contrast, there was no significant difference in pupal weight between the inbred subpopulation with no diet composition change and those fed diets supplemented with arginine, isoleucine, and valine. The pupal weights in those diets supplemented with histidine (*F*_1.202_ = 10.066, *P* < 0.001) and glutamic acid (*F*_1.202_ = 10.037, *P* < 0.001) were 9.23% and 8.10% higher, respectively, than that in those fed a diet with no amino acid supplementation (Fig. 3F). The pupal weights in the inbred populations fed diets supplemented with valine (*F*_1.202_ = 28.818, *P* = 0.904) and isoleucine (*F*_1.202_ = 22.995, *P* = 0.200) were 14.76 ± 0.04 mg and 14.63 ± 0.05 mg, respectively (Supplementary Information Fig. 2C, Fig. 3F). When the inbred populations were fed on a spent diet (i.e. food that had already been eaten for 3 days by larvae of outbred populations), the pupal weight in the inbred population was increased by 1.06% (*F*_1.63_ = 0.472, *P* < 0.05, Fig. 3G). Similarly, the pupal weight in the outbred population was decreased by 6.28% (*F*_1.74_ = 0.853, *P* <0.001) when they were feed on a spent diet of the inbred population (Supplementary Information Fig. 2D, Fig. 3G).

### Maternal inheritance

We compared the phenotypic differences between the inbred and outbred populations from the same female source (Supplementary Information Fig. 3). We did not find maternal effects (i.e. effects of the female source) on the phenotypes of hybridized flies of outbred population. The outbred F♀ × H♂ population had a higher pupal weight (*F*_1.97_ = 381.062, *P* < 0.001) and ovary size (*F*_1.23_ = 6.784, *P* < 0.001) than the F population, while the two inbred populations showed no differences (Supplementary Information Fig. 3A and B). Furthermore, the outbred F♀ × H♂ population had lower survival (*F*_1.5_ = 11.250, *P* < 0.001) and higher fecundity (*F*_1.7_ = 1.627, *P* < 0.001) than the F population (Supplementary Information Fig. 3C and D). Additionally, the outbred H♀ × F♂ population had better performance in terms of pupal weight, ovary size, survival, and fecundity than the inbred H population. As they had the same phenotype, we did not find that outbred populations from the same female source significantly differed in life history parameters.

We even examined the microbiome in the oviposition fluids of female oriental fruit flies. However, the microbiome composition in the oviposition fluids was quite different from that in the intestinal flora (Supplementary Information Fig. 4A and B). Thus, the female source did not affect the microbiome through vertical transmission in oviposition fluids (i.e. that there were no differences between inbred and outbred populations from the same female source).

We also examined maternal effects of inbred population on the intestinal microflora of the hybridized flies of outbred population. The microbiome in intestinal flora of the inbred F population and the outbred F♀ × H♂ population were present in Supplementary Information Fig. 5A and B. Many intestinal bacteria and intestinal fungi were enriched in the outbred F♀ × H♂ population compared with F inbred population (Supplementary Information Fig. 5C and D). Microbiome composition in intestinal flora of the inbred H population and the outbred H♀ × F♂ population were present in Supplementary Information Fig. 6. Finally, we found that *Diutina rugosa* and *Komagataeibacter saccharivorans* were enriched in both outbred H♀ × F♂ and F♀ × H♂ population.

### Transcriptomic differences between inbred and outbred OFFs

Genomic material of the inbred and outbred populations was sequenced on the Illumina platform, and 46.83 × 10^6^, 49.30 × 10^6^, 50.33 × 10^6^, and 49.65 × 10^6^ high-quality sequences were obtained. Each sample generated approximately 7 G of data, with the Q20 value reaching 98%. A total of 24,000 genes and 36,955 transcripts were obtained after assembly. The longest transcript was 16,079 bp, the shortest was 201 bp, and the average length was 1082.85 bp. Most of the transcripts were distributed between 200 and 500 bps, and the length of N50 was 2156 bp.

**Fig. 4:**
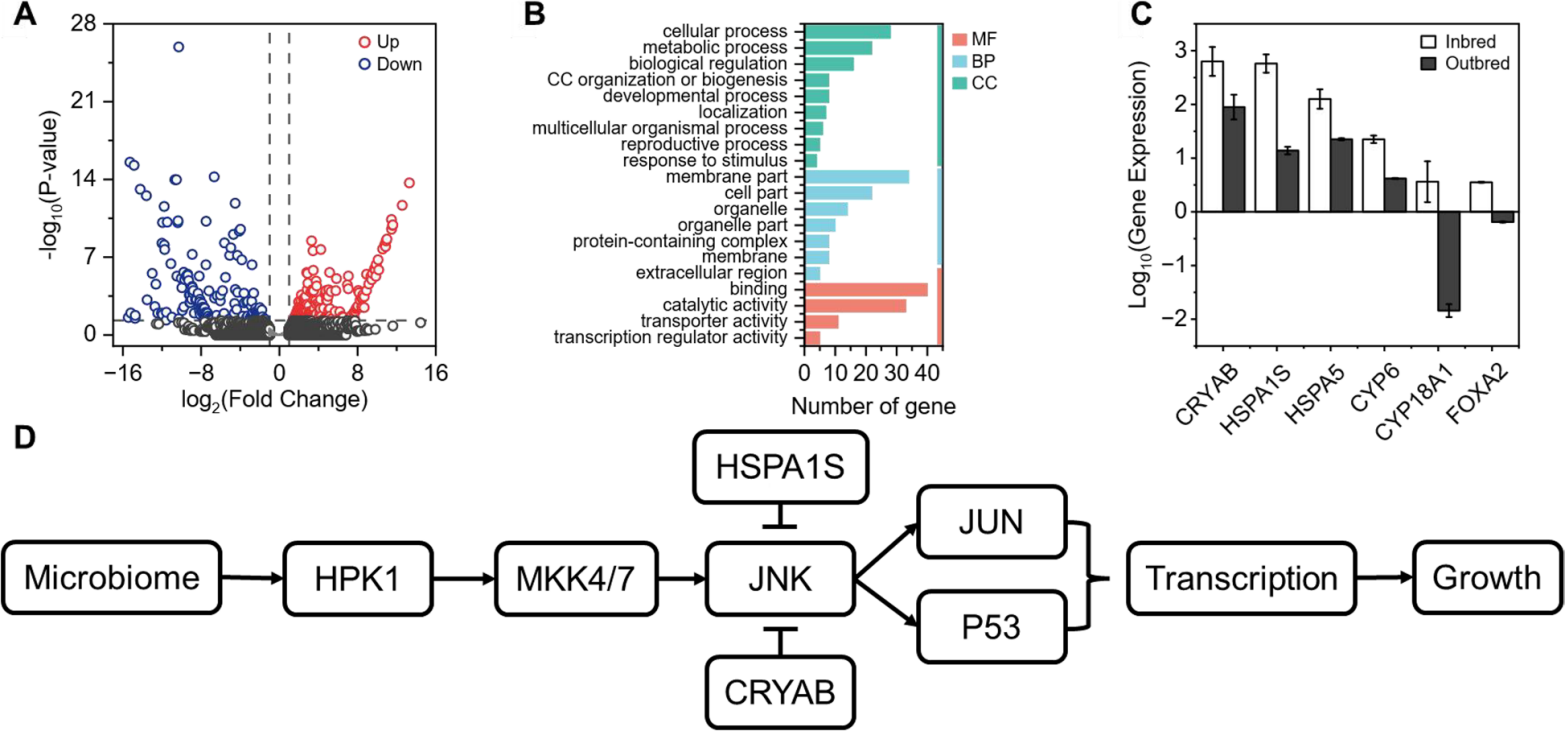
Transcriptomic analyses of the inbred and outbred populations. **A** Volcano map, the horizontal coordinate indicates the change in the gene expression (inbred vs. outbred population) (log2 fold change); the vertical coordinate indicates the significance level (-log10 (*P*-value)). The upregulated genes and downregulated genes are presented by red and green dots, respectively. Grey represents no significant difference. **B** The GO functions of significantly different genes were enriched in the outbred population of *B. dorsalis*. MF stands for molecular function, BP for biological processes and CC for cell components. **C** Significantly downregulated gene expression in outbred populations. **D** Intestinal transcriptome metabolic pathway map

A false discovery rate (FDR) ≤ 0.05 and fold change ≥ 2 were applied to compare unigenes between the inbred and outbred populations. The results showed that 784 unigenes were differentially expressed in the transcriptomes of the inbred and outbred populations, of which 637 unigenes were upregulated and 147 unigenes were downregulated (Fig. 4A).

Thirty-four functional components and 147 downregulated unigenes in the outbred population were obtained based on 309 annotations. Among them, 95 annotations were attributed to molecular function, 110 to biological processes, and 104 to cellular components. In the molecular function category, binding (GO:0005488) (27.21%) and catalytic activity (GO:0003824) (22.45%) were the most enriched. Cellular process (GO:0009987) (19.05%) and metabolic process (GO:0005488) (14.97%) were the most involved biological processes. Cell part (GO:0008152) (23.13%) and membrane part (GO:0044425) (14.97%) were the most upregulated cellular components (Fig. 4B).

Some downregulated differential genes that may affect phenotypic differentiation were screened considering *P* < 0.05. The expression levels of the CRYAB gene in the inbred and outbred populations were 750.15 ± 36.49 and 101.47 ± 47.49, respectively. The expression levels of HSPA1S in the inbred and outbred populations were 617.63 ± 336.22 and 14.04 ± 2.09, respectively. The expression levels of the HSPA5 gene in the inbred and outbred populations were 136.90 ± 51.12 and 22.25 ± 0.98, respectively. The expression levels of the CYP6 gene in the inbred and outbred populations were 22.76 ± 3.58 and 4.18 ± 0.09, respectively. The expression levels of CYP18A1 in the inbred and outbred populations were 4.96 ± 33.13 and 0.02 ± 0.00, respectively. The expression levels of FOXA2 in the inbred and outbred populations were 3.57 ± 0.05 and 0.64 ± 0.01, respectively (Fig. 4C).

Based on the KEGG database of 122 metabolic pathways, 60 downregulated genes were obtained, among which 10 pathways were significantly enriched in the outbred population (*FDR* ≤ 0.05). The immune system pathway, including the antigen processing and presentation pathway, had the most significant differences between the two populations (*P* = 0.0019).

Additionally, the longevity regulating pathway-multiple species pathway (*P* = 0.0021) and protein processing in the endoplasmic reticulum pathway (*P* = 0.0022) and the MAPK signalling pathway (*P* = 0.0033) showed significant differences. Thus, the expression of the HSPA1S gene in the MAPK pathway was downregulated, which led to upregulation of the JNK pathway. Additionally, the CYP18A1 gene, which regulates the insect hormone synthesis pathway, namely, the ecdysone pathway, was significantly downregulated, but the pathway was not significantly enriched (Table 1, Fig. 4D).

**Table 1:** KEGG enrichment pathways and descriptions

Pathway ID	Description	***p***-**value**
map04612	Antigen processing and presentation	0.0019
map04213	Lifespan regulation pathway — multiple species	0.0021
map04141	Protein processing in endoplasmic reticulum	0.0022
map03040	Spliceosome	0.039
map04010	MAPK signalling pathway	0.045

## Discussion

### Effects of genetic diversity on the phenotype of the OFF

Heterosis refers to the phenomenon that the offspring of a hybrid are superior to their parents in terms of body size, reproductive survival time, and reproductive capacity [44]. The pupal weight, adult biomass, and reproductive capacity in offspring of hybrids were significantly higher than those with the parental phenotype, and the rate of body weight increase in *F*_1_ hybrids was also higher than that in their parents [45]. A Hong Kong oyster indoor seeding study revealed that the hybrid population had a significant survival advantage over the self-crossing population [46]. Heterosis exists in the hybrid offspring of horse-donkey pairs, and the offspring are characterized by strong disease resistance, higher coarse feed tolerance, and a longer lifespan [47]. After crossing purple scallops and Gulf scallops, the fertilization rate of the hybrid scallops was higher than that of the self-breeding population [48]. In this study, it was found that the outbred population of *B. dorsalis* had advantages such as a higher pupal weight, survival rate, and egg quantity, which was consistent with the results of previous studies. It was also found that hybridization had no significant effect on the development duration of larvae and pupae [47, 49].

### Effects of the intestinal microbiota on OFFs

Changes in amino acid compositions led to changes in the microbiome composition [50, 51]. Therefore, differences in intestinal microbiota compositions between the outbred and inbred populations of OFFs were due to differences in amino acid concentrations. Among the outbred and inbred populations, the fungal species with the highest degree of differentiation was *D. rugosa* in Saccharomycetales, and the bacterial species with the highest degree of differentiation was *K. saccharivorans* in *Lactobacillus*. Moreover, the concentrations of histidine, glutamic acid, and arginine in the intestinal tract in the outbred population were significantly higher than those in the intestinal tract in the inbred population. After feeding diets supplemented with amino acids, the intestinal microbiota composition changed in the populations. The intestinal microbiota compositions in the inbred and outbred populations were also altered by exchanging diets between populations. After diet exchange, the pupal weight in the inbred population increased, and that in the outbred population decreased. The results also showed that the increase in pupal weight in the outbred population of the OFF was caused by a change in the microbial environment after the conversion of amino acids in the intestine.

### Effects of the insect transcriptome on genetic diversity

MAPK, short for mitogen-activated protein kinases, is a group of evolutionarily conserved silk/threonine protein kinases that are activated by a series of extracellular stimulation signals and mediate signal transmission from the cell membrane to the nucleus. They regulate many physiological activities, such as inflammation, apoptosis, carcinogenesis, and the metastasis of tumour cells [52]. JNK, also known as stress-activated protein kinase (SAPK), is another subclass of the MAPK (mitogen-activated protein kinase) signalling pathway in mammalian cells. The MAPK-JNK pathway can be activated by various environmental stressors, inflammatory cytokines, growth factors, and CPCR agonists. Stress response signals are transmitted to the targets of this cascade by small molecule GTp-enzymes in the RHO family. JNK transport into the nucleus can regulate the activity of a variety of transcription factors and then mediate the transcriptional activation of JUN, ELK1, P53, etc., which play important roles in various physiological and pathological processes, such as the cell cycle, reproduction, apoptosis, and cell stress [53, 54]. In this study, it was found that the HSPA1S gene was significantly downregulated and enriched in the MAPK pathway. Combined with the observation results from the differential phenotypes, it is believed that downregulated HSPA1S upregulated the JNK gene, which then affected the growth and development of the outbred population. Therefore, the pupal weight in the outbred population increased. Increases in fecundity and other indicators were regulated by the JNK-MAPK pathway, consistent with the results of previous studies [55]; moreover, the HSPA1S gene was also enriched in the pathway of immune function. In this study, it was also found that the CYP18A1 gene was significantly downregulated and enriched in insect synthetic hormones, and the decrease in insect ecdysone could have caused the increase in insect body weight [56].

### Mechanisms by which hybridization promotes invasion

Population mixing has the potential to promote species invasion by creating unique opportunities for positive genetic interactions between previously isolated alleles and the adaptive evolution of new genotypes, leading to improved fitness in hybrid populations. These mechanisms directly contribute to colonization by nonnative species and the evolution of particularly invasive new genotypes in the case of interspecies hybridization, and the potential for interspecies hybridization to yield these benefits is widespread [17]. There are several nonmutually exclusive mechanisms that may produce the following positive fitness effects due to either genetic interactions between new combinations of alleles within individuals or increased genetic diversity resulting from different population combinations [17, 57, 58]: the genetic remedial effect, superdominant effect, epistatic effect, complementary effect, and evolutionary remedial effect. The continued spatial expansion of the OFF due to the domestic trade of fruits and vegetables increases the risk of outbreeding among individuals originating from multiple populations during invasion processes. Sequencing (e.g. 16 srRNA) of intestinal flora has shown that gut communities of insect hosts are typically simple (often dominated by a few key taxa) with the majority of the gut microbiome composed of non-specialist microbes [59, 60]. Thus, the insect hosts tend to be newly acquired a beneficial gut symbiont from the external environment every generation [61, 62]. Additionally, some studies have shown that microbes in organisms may be a newly discovered mechanism promoting hybrid invasion [28]. In this study, a novel mechanism of invasiveness in hybridized OFFs was proposed, that is, intestinal microbial-mediated hybridization induced phenotypic changes and promoted the invasion of *B. dorsalis* [63].

## Conclusion

Intraspecific hybridization of IAS has the potential to contribute to enhanced invasiveness and genetic resiliency to rapidly adapt to newly invaded habitats [64, 65]. Our study revealed difference in the life history traits of outbred and inbred populations of the OFF. Both the survival curves and oviposition rates of outbred populations were increased by population mixing. We also found that composition changes in the gut microbiota caused an increase in essential amino acid concentrations, which led to a shift in HSPA1S transcription. We found that downregulation of the HSPA1S gene in the intestinal tract of outbred populations of OFFs may affect longevity and body weight gain in outbred populations through upregulation of the JNK-MAPK pathway. It can also affect the cell apoptosis and reproductive functions in insects to enhance survival and fecundity in outbred populations. We highlight that these intraspecific hybrids have the potential to produce a variety of unique genetic combinations that could result in greater success among outbred populations in their invaded range, potentially facilitating the colonization of invasive species [66].We conducted an exploratory study on OFF invasiveness, revealed phenotypic differentiation between inbred and outbred populations, and proposed a physiological mechanism of intestinal microbiota-mediated outbreeding success, promoting the invasion of the oriental fruit fly. The physiological mechanism of hybrid invasion promotion provides a new avenue for the study of hybrid invasion [67, 68].

### Supplementary Information

**Additional file 1:** **Supplementary Information for results.** **Supplementary Information Fig. 1.** Differences in invasiveness between the original (Fujian and Hainan) and mixed outbred populations (invasive population) of *B. dorsalis*. A, pupal weight; B, ovary size at 15 d after emergence; C, number of eggs laid per female per day; D, hatching rate. **Supplementary Information Fig. 2.** The effects of amino acid and intestinal microbe on body weight of the oriental fruit fly. A, Cephalo-pharyngeal bone length in inbred and outbred populations of *B. dorsalis*. B, Food intake of B. dorsalis inbred population and outbred population. C, pupal weight of inbred populations supplemented with different amino acids. D, pupa weight after feed exchange between inbred and outbred populations. **Supplementary Information Fig. 3.** Phenotypic differences between inbred and outbred populations of *B. dorsalis*. A, pupal weight; B, ovary size at 15 d after adult emergence; C, survival fraction; D, fecundity. Asterisks indicate significant differences (*,*p*<0.05; **, *p*<0.01, ***, *p*<0.001), and *ns* indicates no significant differences. **Supplementary Information Fig. 4.** Species compositions of the microbiomes of the intestinal flora and oviposition fluids in F populations of B. dorsalis. A, bacterial composition and relative abundance; B, fungal composition and relative abundance. The inner circle represents the oviposition fluids, and the outer circle represents the intestinal flora. **Supplementary Information Fig. 5.** Species compositions of the microbiomes of the intestinal flora in the inbred F and the outbred F♀×H♂ populations of *B. dorsalis*. A, bacterial composition and relative abundance; B, fungal composition and relative abundance. The inner circle represents the inbred F population, and the outer circle represents the outbred F♀×H♂ population. LEfSe diagram of intestinal bacteria (C) and fungi (D) between inbred F and outbred F♀×H♂ populations. **Supplementary Information Fig. 6.** Species compositions of the microbiomes of the intestinal flora in the inbred H and the outbred H♀×F♂ populations of *B. dorsalis*. A, bacterial composition and relative abundance; B, fungal composition and relative abundance. The inner circle represents the inbred H population, and the outer circle represents the outbred H♀×F♂ population. LEfSe diagram of intestinal bacteria (C) and fungi (D) between inbred H and outbred H♀×F♂ populations. **Supplementary Information Table 1.** The artificial diet of *Bactrocera dorsalis*. **Supplementary Information Table 2.** Life table parameters of the inbred and the outbred populations of*B. dorsalis.* **Supplementary Information for methods.** Microbiome determination and analysis: DNA extraction and sequencing. Analysis of microbiome data. Diet exchange experiments.

## Data Availability

The sequencing dataset for transcriptome has been submitted to the National Center for Biotechnology Information; accession is SRR21206475-SRR21206478 (SRP394084). The sequencing dataset for gut microbes has been submitted to the National Center for Biotechnology Information; accession is SRR21275060-SRR21275070 (SRP394415).
