# Supplementary material for: Population mixing mediates the intestinal flora composition and facilitates invasiveness in a globally invasive fruit fly
Source: Microbiome. 2023 Sep 28;11:213. doi: 10.1186/s40168-023-01664-1 (PMC10538247; doi:10.1186/s40168-023-01664-1)
Supplement: Supplementary file 2 — Additional file 1: Supplementary Information for results. Supplementary Information Fig. 1. Differences in invasiveness between the original (Fujian and Hainan) and mixed outbred populations (invasive population) of B. dorsalis. A, pupal weight; B, ovary size at 15 d after emergence; C, number of eggs laid per female per day; D, hatching rate. Supplementary Information Fig. 2. The effects of amino acid and intestinal microbe on body weight of the oriental fruit fly. A, Cephalo-pharyngeal bone length in inbred and outbred populations of B. dorsalis. B, Food intake of B. dorsalis inbred population and outbred population. C, pupal weight of inbred populations supplemented with different amino acids. D, pupa weight after feed exchange between inbred and outbred populations. Supplementary Information Fig. 3. Phenotypic differences between inbred and outbred populations of B. dorsalis. A, pupal weight; B, ovary size at 15 d after adult emergence; C, survival fraction; D, fecundity. Asterisks indicate significant differences (*,p<0.05; **, p<0.01, ***, p<0.001), and ns indicates no significant differences. Supplementary Information Fig. 4. Species compositions of the microbiomes of the intestinal flora and oviposition fluids in F populations of B. dorsalis. A, bacterial composition and relative abundance; B, fungal composition and relative abundance. The inner circle represents the oviposition fluids, and the outer circle represents the intestinal flora. Supplementary Information Fig. 5. Species compositions of the microbiomes of the intestinal flora in the inbred F and the outbred F♀×H♂ populations of B. dorsalis. A, bacterial composition and relative abundance; B, fungal composition and relative abundance. The inner circle represents the inbred F population, and the outer circle represents the outbred F♀×H♂ population. LEfSe diagram of intestinal bacteria (C) and fungi (D) between inbred F and outbred F♀×H♂ populations. Supplementary Information Fig. 6. Species compositi [file 40168_2023_1664_MOESM1_ESM.docx]

**Supplementary Information for**

Population mixing mediates metabolism and the intestinal flora composition and facilitates invasiveness in a globally invasive fruit fly

Yidan Wang^1^, Zhihong Li^1^, Zihua Zhao^1,*^

^1^ Department of Plant Biosecurity, College of Plant Protection, China Agricultural University, Beijing 100193, China

Email: zhzhao@cau.edu.cn

Supplementary Information for results

We found that the mixed outbred population had significantly higher pupal weight, fecundity, and hatching rates than the original population in the mangos. The performance of the mixed outbred population indicated that it would have higher invasiveness than the original F and H population, which was also verified in our experiment. The mixed outbred population had higher pupal weight and ovary size than both the original F and H populations. Additionally, the number of eggs laid per female per day and hatching rate were higher in the mixed outbred population than in the original F and H population.


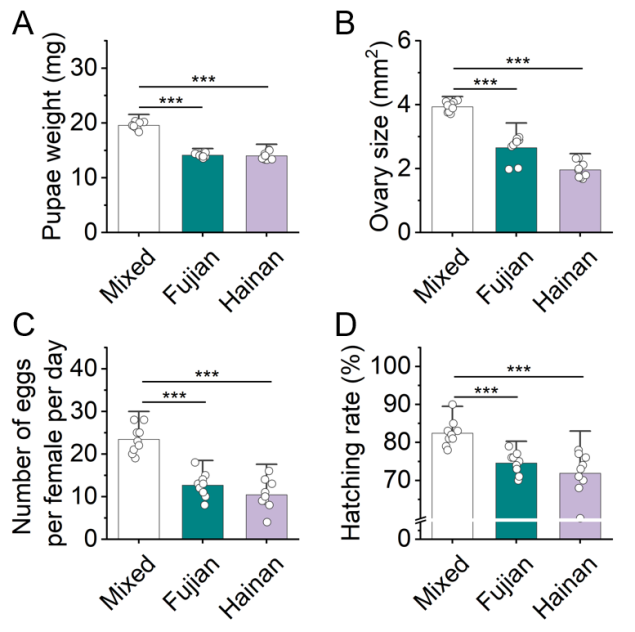


**Supplementary Information Figure 1**. Differences in invasiveness between the original (Fujian and Hainan) and mixed outbred populations (invasive population) of *B. dorsalis*. A, pupal weight; B, ovary size at 15 d after emergence; C, number of eggs laid per female per day; D, hatching rate.


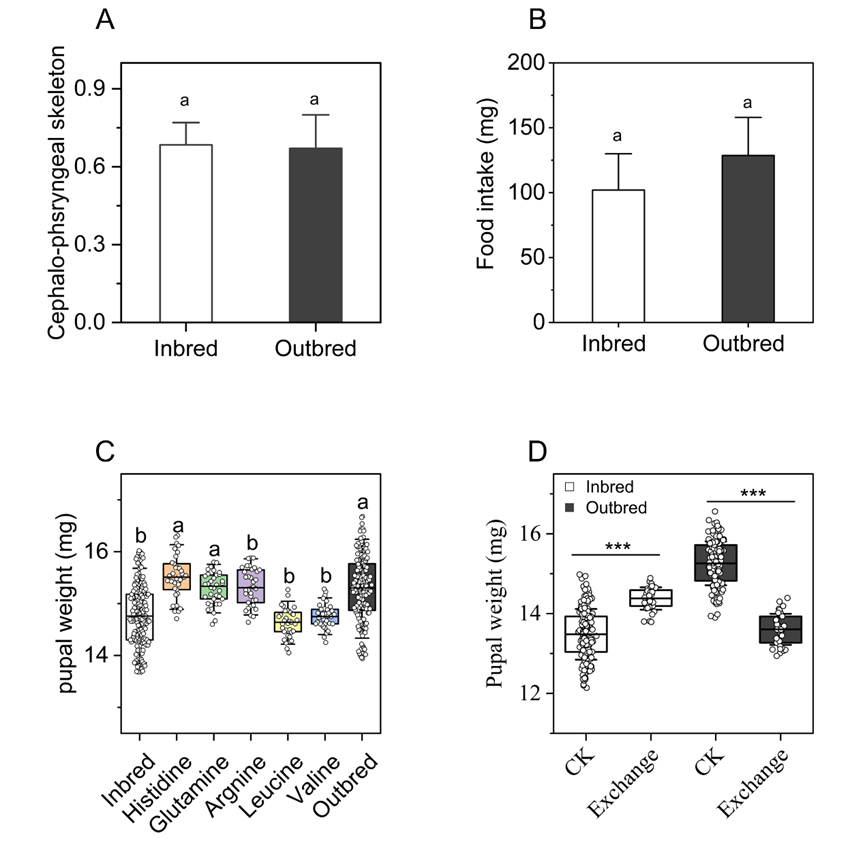


**Supplementary Information Figure 2.** The effects of amino acid and intestinal microbe on body weight of the oriental fruit fly. A, Cephalo-pharyngeal bone length in inbred and outbred populations of *B. dorsalis*. B, Food intake of B. dorsalis inbred population and outbred population. C, pupal weight of inbred populations supplemented with different amino acids. D, pupa weight after feed exchange between inbred and outbred populations.


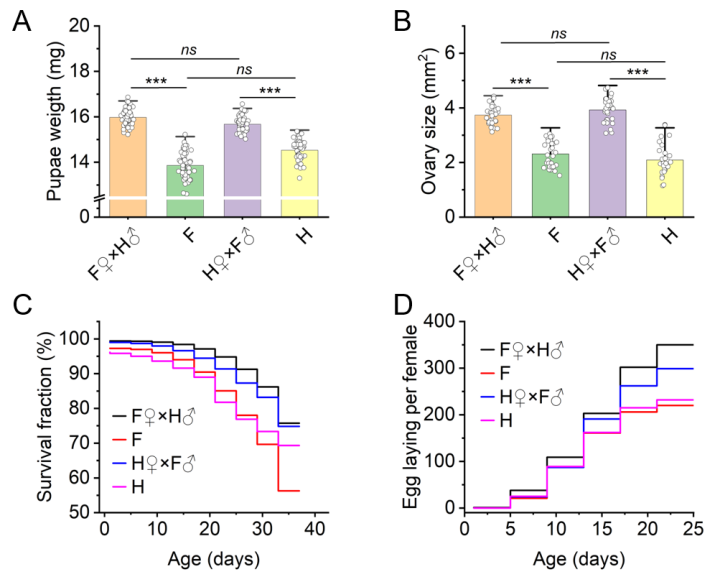


**Supplementary Information Figure 3.** Phenotypic differences between inbred and outbred populations of *B. dorsalis*. A, pupal weight; B, ovary size at 15 d after adult emergence; C, survival fraction; D, fecundity. Asterisks indicate significant differences (*, p<0.05; **, p<0.01, ***, p<0.001), and *ns* indicates no significant differences.

We compared the intestinal microflora between inbred and outbred populations from the same female source. We assumed that the intestinal microflora of the oriental fruit fly was a result of vertical transmission from parental flies. If so, the microbiome in the oviposition fluids of female flies should have similar or shared components with the intestinal flora. In fact, we examined the microbiome in the oviposition fluids of female oriental fruit flies. However, the microbiome composition in the oviposition fluids was quite different from that in the intestinal flora (Supplementary Information Figure 3A and B). Only three bacteria (*Lactococcus* sp., *Paenibacillus* sp., and *Paracoccus* sp.) in oviposition fluids were enriched in the intestinal flora of the F population. However, all three of these bacteria also existed in the gut of H population, which indicates that *Lactococcus* sp., *Paenibacillus* sp., and *Paracoccus* sp. are basic shared bacteria among all populations of the oriental fruit fly. We did not find the specific microbe transmitted by female source. Thus, we proposed that female source did not affect the microbiome through vertical transmission in oviposition fluids (i.e., that there were no differences between inbred and outbred populations from the same female source).


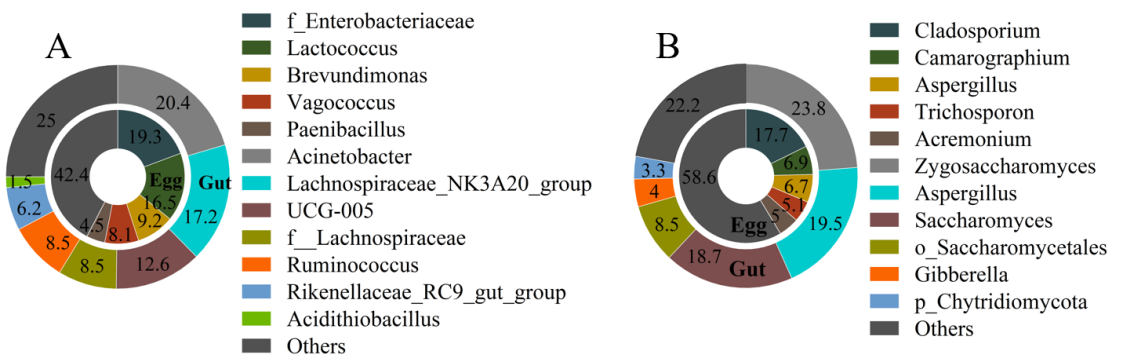


**Supplementary Information Figure 4.** Species compositions of the microbiomes of the intestinal flora and oviposition fluids in F populations of B. dorsalis. A, bacterial composition and relative abundance; B, fungal composition and relative abundance. The inner circle represents the oviposition fluids, and the outer circle represents the intestinal flora.

We also examined maternal effects of inbred population on the intestinal microflora of the hybridized flies of outbred population. Many species in intestinal flora were present in both the inbred F population and outbred F♀×H♂ population (Supplementary Information Figure 4A and B).

7 species of intestinal bacteria were enriched in the outbred F♀×H♂ population compared with H inbred population (Supplementary Information Figure 4C). Also, 10 species of intestinal fungi were enriched in the outbred F♀×H♂ population compared with F inbred population (Supplementary Information Figure 4D).


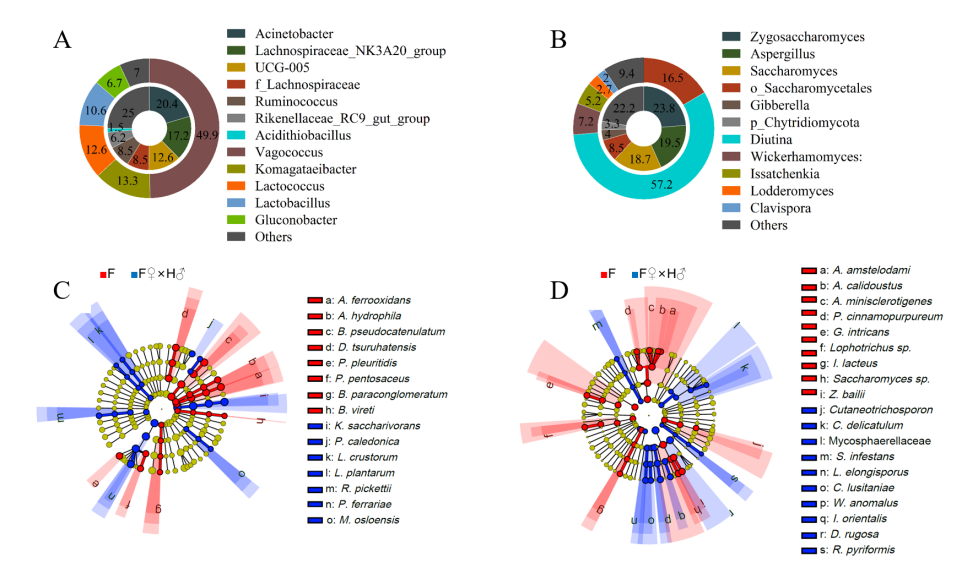


**Supplementary Information Figure 5**. Species compositions of the microbiomes of the intestinal flora in the inbred F and the outbred F♀×H♂ populations of *B. dorsalis*. A, bacterial composition and relative abundance; B, fungal composition and relative abundance. The inner circle represents the inbred F population, and the outer circle represents the outbred F♀×H♂ population. LEfSe diagram of intestinal bacteria (C) and fungi (D) between inbred F and outbred F♀×H♂ populations.

Microbiome composition in intestinal flora were present in both the inbred H population and outbred H♀×F♂ population (Supplementary Information Figure 5A and B). 8 species (e.g. *Komagataeibacter saccharivorans*) of intestinal bacteria were enriched in the outbred F♀×H♂ population compared with the inbred H population (Supplementary Information Figure 5C). Also, 8 species (e.g. *Cladosporium delicatulum* and *Diutina rugosa*) of intestinal fungi were enriched in the outbred F♀×H♂ population compared with the inbred F population (Supplementary Information Figure 5D). We found that both *Diutina rugosa* and *Komagataeibacter saccharivorans* were enriched in both outbred H♀×F♂ and F♀×H♂ population.

Due to the higher phenotypic performance of both the F♀×H♂ and H♀×F♂ populations, we propose that coenrichment of *Diutina rugosa* and *Komagataeibacter saccharivorans* is a potential mechanism that enhances invasiveness in outbred populations. These microbiome shifts, which occurred in the F♀×H♂ and H♀×F♂ outbred population, caused metabolic changes and led to phenotypic differences. We did not find female-specific phenotypic differences in inbred and outbred populations from the same female source. Instead, we found that both *Diutina rugosa* and *Komagataeibacter saccharivorans* were significantly enriched in all outbred populations, which had higher genetic heterozygosity.


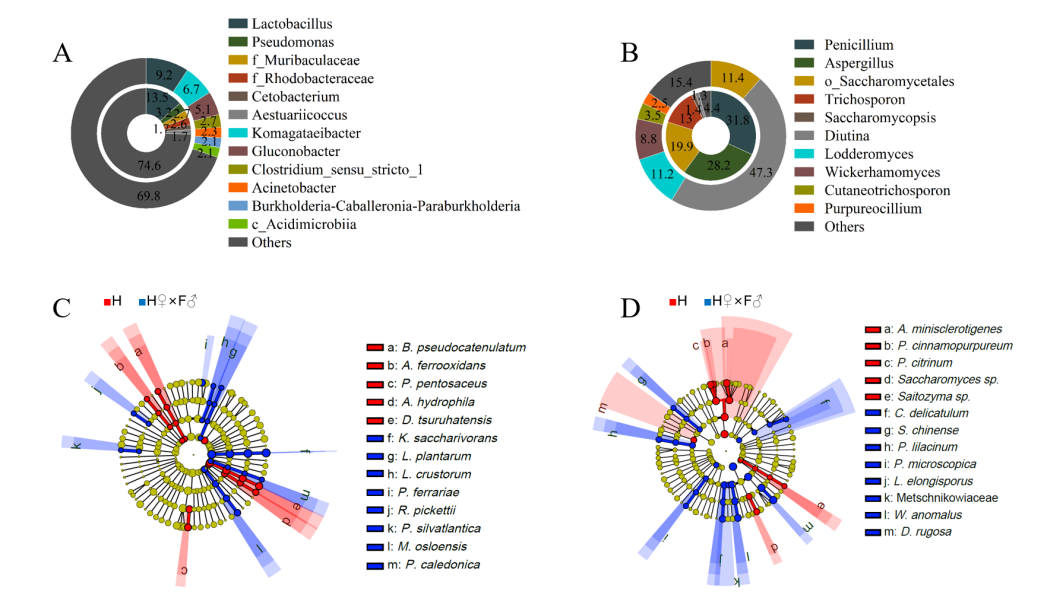


**Supplementary Information Figure 6**. Species compositions of the microbiomes of the intestinal flora in the inbred H and the outbred H♀×F♂ populations of *B. dorsalis*. A, bacterial composition and relative abundance; B, fungal composition and relative abundance. The inner circle represents the inbred H population, and the outer circle represents the outbred H♀×F♂ population. LEfSe diagram of intestinal bacteria (C) and fungi (D) between inbred H and outbred H♀×F♂ populations.

**Supplementary Information Table 1**. The artificial diet of *Bactrocera dorsalis*

| Artificial diet for larva  （Liu et al.,2017） | |  | Artificial die for adults  （Cui, 2021） | |
| --- | --- | --- | --- | --- |
| Component | Content |  | Component | Content |
| Sucrose | 125g |  | Sucrose | 150g |
| Beer yeast | 31g |  | Soybean peptone | 50g |
| Wheat bran | 325g |  |  |  |
| Sorbic acid | 1g |  |  |  |
| Methyi-p-hydroxybenzoate | 0.6g |  |  |  |
| L-ascorbic acid | 0.5g |  |  |  |
| H_2_O | 600ml |  |  |  |

**Supplementary Information Table 2**. Life table parameters of the inbred and the outbred populations of *B. dorsalis.*

| Parameters  Population | R_0_ | r_m_ | λ | T | DT |
| --- | --- | --- | --- | --- | --- |
| H | 316.08±15.02b | 0.33±0.00a | 1.39±0.01a | 17.43±0.19b | 2.10±0.03a |
| F | 262.45±13.29b | 0.32±0.01a | 1.38±0.01a | 17.36±0.25b | 2.16±0.04a |
| F♀×H♂ | 409.43±17.17a | 0.33±0.01a | 1.39±0.01a | 18.02±0.41a | 2.06±0.07b |
| H♀×F♂ | 271.88±22.87b | 0.30±0.01b | 1.34±0.01b | 19.13±0.36a | 2.28±0.04a |
| Inbred | 289.27±13.01a | 0.33±0.00a | 1.38±0.00a | 17.40±0.15b | 2.13±0.02a |
| Outbred | 333.01±27.90a | 0.32±0.01a | 1.37±0.01a | 18.32±0.29a | 2.19±0.04a |

R_0_ represents the net appreciation rate of effective population, r_m_ represents the effective intrinsic growth rate, λ represents the effective weekly growth rate, T represents the average period of generations, DT represents the effective population doubling time.

**Supplementary Information for methods**

***Microbiome determination and analysis***

***DNA extraction and sequencing***

Genomic DNA was extracted from the tissue samples and detected by 1% agarose gel electrophoresis, and after passing the DNA quality check, specific primers were synthesised for PCR amplification according to the specified sequencing regions. The PCR products were detected by 2% agarose gel electrophoresis and purified by gel cutting using the AxyPrepDNA Gel Recovery Kit (AXYGEN). The PCR products were quantified by using the QuantiFluor™ -ST Blue Fluorescence Quantification System (Promega) with reference to the preliminary quantification results of electrophoresis, and then mixed in the appropriate proportions according to the sequencing volume required for each sample. Miseq libraries were constructed and sequenced, and the results were used for subsequent microbiome analysis.

***Analysis of microbiome data***

The PE reads from the Miseq series were first assembled by overlap relationships, and the quality of sequence information was quality controlled and screened. The results of the OTU clustering analysis will be used to obtain the corresponding data on the diversity of the community, including the diversity structure. The results of the taxonomic analysis are also used to understand the taxonomic characteristics of each sample at each taxonomic level. Biometric analysis is required to understand which bacteria are present in the data and the number of sequences of each bacterium in the data, as well as the relative abundance of each species, and thus to observe the composition of biological populations at the taxonomic level of the data. The species richness data obtained for the biomes were used to evaluate the significance level of differences in species richness using LEfSe by conducting hypothesis tests on the species between the different groups of microbial populations in a statistical way to obtain the different species that differ significantly between the inbred and distant groups. The data analysis were performed using the online platform of Majorbio Cloud Platform ([www.majorbio.com](http://www.majorbio.com)).

***Diet exchange experiments***

A certain number of eggs were reared from the inbred and the outbred population, with replicates of each population and 300 eggs picked from each replicate. On day 5 after eggs hatch, all larvae from both the inbred and the outbred populations were exchanged the spent diets. We fed larvae of outbred populations a spent diet (i.e., food that had already been eaten for 3 d by larvae of inbred populations). Similarly, the larvae of inbred populations were fed a spent diet that had been eaten for 3 d by larvae of outbred populations. We exchanged the spent diets of the outbred populations and inbred population to examine the influence of gut microbiome exchange on the life history of flies.

Three replicates were carried out for each group. After all larvae had developed into mature larvae, all larvae were picked up and placed in a box with moist sterile sand and left to pupate. On the 5th day after pupation, the pupae were sieved out, washed with deionised water and left on gauze for 24 hours at room temperature. The dead insects were removed from the weighing process.
